# BioByGANS: biomedical named entity recognition by fusing contextual and syntactic features through graph attention network in node classification framework

**DOI:** 10.1186/s12859-022-05051-9

**Published:** 2022-11-22

**Authors:** Xiangwen Zheng, Haijian Du, Xiaowei Luo, Fan Tong, Wei Song, Dongsheng Zhao

**Affiliations:** 1 Academy of Military Medical Sciences, Beijing, 100039 China; 2 Beijing MedPeer Information Technology Co., Ltd, Beijing, 102300 China

**Keywords:** Biomedical named entity recognition, Text mining, BioBERT, SpaCy, Graph attention network, Contextual features, Syntactic features

## Abstract

**Background:**

Automatic and accurate recognition of various biomedical named entities from literature is an important task of biomedical text mining, which is the foundation of extracting biomedical knowledge from unstructured texts into structured formats. Using the sequence labeling framework and deep neural networks to implement biomedical named entity recognition (BioNER) is a common method at present. However, the above method often underutilizes syntactic features such as dependencies and topology of sentences. Therefore, it is an urgent problem to be solved to integrate semantic and syntactic features into the BioNER model.

**Results:**

In this paper, we propose a novel biomedical named entity recognition model, named BioByGANS (**BioB**ERT/SpaC**y**-**G**raph **A**ttention **N**etwork-**S**oftmax), which uses a graph to model the dependencies and topology of a sentence and formulate the BioNER task as a node classification problem. This formulation can introduce more topological features of language and no longer be only concerned about the distance between words in the sequence. First, we use periods to segment sentences and spaces and symbols to segment words. Second, contextual features are encoded by BioBERT, and syntactic features such as part of speeches, dependencies and topology are preprocessed by SpaCy respectively. A graph attention network is then used to generate a fusing representation considering both the contextual features and syntactic features. Last, a softmax function is used to calculate the probabilities and get the results. We conduct experiments on 8 benchmark datasets, and our proposed model outperforms existing BioNER state-of-the-art methods on the BC2GM, JNLPBA, BC4CHEMD, BC5CDR-chem, BC5CDR-disease, NCBI-disease, Species-800, and LINNAEUS datasets, and achieves F1-scores of 85.15%, 78.16%, 92.97%, 94.74%, 87.74%, 91.57%, 75.01%, 90.99%, respectively.

**Conclusion:**

The experimental results on 8 biomedical benchmark datasets demonstrate the effectiveness of our model, and indicate that formulating the BioNER task into a node classification problem and combining syntactic features into the graph attention networks can significantly improve model performance.

## Background

Biomedical named entity recognition (BioNER), which is a subdivision of named entity recognition (NER) [1], aims to identify the mention of biomedical named entities such as genes, proteins, diseases, drugs, species, etc. in texts [2, 3]. Automatically and accurately extracting biomedical named entities is the prerequisite for extracting biomedical knowledge from unstructured texts into structured formats, which helps researchers track and summarize the knowledge contained in the extensive scientific literature in a timely manner.

With the in-depth study of deep learning [4], deep learning methods have been widely used in the field of natural language processing (NLP). BioNER, modeled as a sequence labeling problem [5], can then be solved end-to-end by deep learning methods, which avoids manual feature engineering and improves the performance to a certain extent. However, a major problem is the lack of large-scale high-quality annotated training data. In addition, the sequence labeling framework underutilizes and fails to explicitly exploit the topological information of language to some extent.

**Fig. 1:**
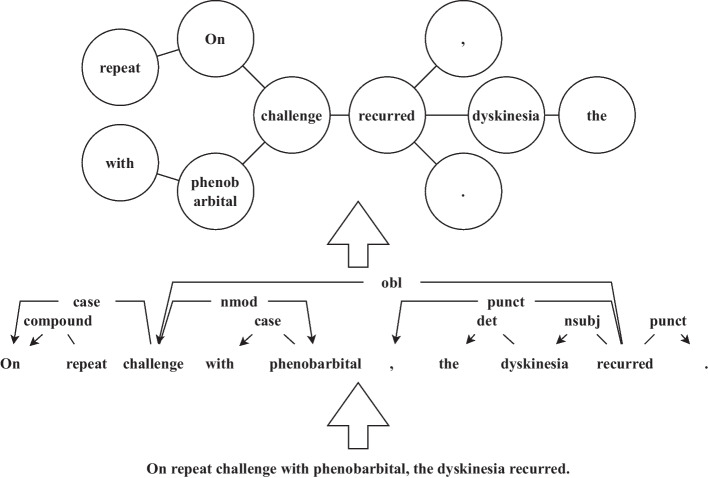
An example of using the graph to model the topology of a sentence. The topology of a sentence is a graph instead of a sequence. As shown in the figure, challenge is neighbor of recurred in the sentence graph, though challenge is far away from recurred in the sequence. There exists 2 biomedical NEs in the sentence, which serve as subject and object structure respectively. The sentence graph reflects the correlation path and indicates the syntactic relationship between the 2 entities, which helps to implement the NER task

Pre-trained models, such as Word2Vec [6], ELMo [7], and BERT [8], first use a self-supervised learning strategy to learn distributed representations of words in the large-scale unlabeled corpus and then perform finetune according to downstream tasks. Among them, BERT, a state-of-the-art (SOTA) regressive language model, provides an important foundation for various NLP downstream tasks. The pre-training strategy is also implemented in the biomedical field, and models such as BioWord2Vec [9], BioELMo [10], and BioBERT [11], have already been proposed. However, syntactic features of texts including part of speeches, constituencies, and dependencies [12], which should play an important role in NLP tasks, are currently under-considered by pre-trained models. As shown in Fig. 1, a sentence graph is built based on syntactic features. Different from the sequence structure, the graph structure can better define the semantic distance between words, which may help to implement the NLP tasks.

In this paper, inspired by the topological information of language, we use a graph to model a sentence. Using a graph to model sentences can introduce more topological information of language, and make the model focus more on topological distances rather than sequential distances between words. Then, we formulate the BioNER task as a node classification problem and propose an end-to-end model, BioByGANS, which integrates both contextual and syntactic features of texts. Among them, contextual features include but not limited to semantic, position and morphological information, and syntactic features include part of speech and dependency information. First, we use periods to segment sentences and spaces and symbols to segment words. Second, contextual features and syntactic features of a sentence are encoded via a pre-trained model BioBERT and a NLP tool SpaCy [13] respectively. A graph attention network (GAT) [14] is then used to generate representations according to the topology of the sentence and contextual features. Last, a softmax function is used to calculate the probabilities and get the results. We evaluate our model on 8 biomedical benchmark datasets, and the experimental results show that BioByGANS outperforms the current SOTA models. Our contributions are summarized as follows:

1. A novel word representation which fuses contextual features (BioBERT) and syntactic features including part of speeches and dependencies (SpaCy), and is further optimized via graph attention network.
2. A novel method of formulating BioNER task as a node classification problem based on sentence-level topological features.

The remainder of this paper is organized as follows. In chapter II, related work of BioNER is presented. In chapter III, the proposed model is introduced in detail. And chapter IV presents the designed experiments and results. Finally, the paper is concluded in chapter V.

## Related work

IN this chapter, the recent progress of BioNER is first introduced. The preliminary knowledge of components in our model, including text distribution representation, syntactic features, and graph neural networks, is then presented.

### Recent progress of BioNER

Traditional methods for BioNER include rule-based [15], dictionary-based [16], and machine learning methods [17, 18]. However, these methods rely heavily on hand-crafted work. Furthermore, the above hand-crafted work is entity-specific, which has poor robustness and cannot solve the problem of polysemy and out of vocabulary (OOV) [19].

Recently, deep learning based methods have already been widely used in BioNER field. The end-to-end strategy avoids manual feature engineering and improves the performance of such models. [20–23] used long short-term memory (LSTM) networks, and [24–26] used conditional random field (CRF) to recognize biomedical entities. [27] realized BioNER based on a semi-Markov classifier and a dictionary-based postprocessing method. [20] and [28–31] implemented the task based on BiLSTM-CRF framework. Specifically, [20] proposed a document-level attention coefficient to transmit features between sentences and realizes NER for chemicals. [28] used a character-level BiLSTM and a word-level BiLSTM respectively to obtain morphologic features and contextual features of words. [29] made a more accurate prediction by exchanging information from single-task models for genes, chemicals, and diseases respectively. [30] built a dictionary based on the disease ontology and then constructs a document-level attention layer by using the dictionary. [31] used a multi-task learning strategy, and got improved by sharing parameters between tasks. [32] is an improved BioNER model by leveraging syntactic information through a key-value memory network.

Pre-trained language models have also been applied in BioNER, and got SOTA performances. BioBERT [11], a model further trained in abstracts and full texts of biomedical publications from PubMed and PMC on the foundation of BERT, achieved high performance based on the pretraining-finetuning strategy. [33, 34], based on BioBERT, implemented BioNER by leveraging the strategy of machine reading comprehension and multi-task learning respectively. BioELECTRA [35], a pre-trained model based on the generative adversarial strategy, is a lighting pre-trained model in biomedical NLP field, which can also be applied in BioNER task.

### Text distributed representation

Distributed representation of texts is the basis of using deep learning methods to realize the downstream tasks of NLP. Language models [36] utilize probability distribution to quantitatively model natural language, and one important method is to map natural language into vector space [37]. Text distributed representation goes through the following 3 stages.

Word embedding methods based on statistics include one-hot encoding, bag of words [38], and TF-IDF [39]. The above methods, however, neglect the word order and contextual characteristics of the text to some extent. In addition, they cannot deal well with the large-scale corpus, nor can they calculate the similarity between words.

Word2Vec [6] employs the continuous bag of words (CBOW) and Skip-Gram, and obtains the distributed representation based on the local context. FastText [40] obtains the morphological features of words based on the word-level sliding windows, which solves the OOV problem. Glove [41] generates the word representation based on a co-occurrence matrix to integrate the global information into the representation. However, the above representations are fixedly stored, which ignores the different contexts of words, and fails to solve the problem of polysemy.

Using dynamic contextual word distributed representations including CoVe [42], ELMo [7], GPT [43], and BERT [8] to solve NLP problems has become a trend because the representation can be adjusted with the change of context. ELMo [7], a pre-trained model based on Bi-LSTM [44], uses the next word prediction task to learn the word representation. BERT [8] generates the representation based on the transformer [45], and obtains sentence-level features through masked language modeling (MLM) and next sentence prediction (NSP).

### Syntactic features

The part of grammar that presents a speaker’s knowledge of sentences and their structures is called syntax, which is the sentence patterns of language [12]. The syntax is one of the important research objects and the important characteristics of downstream tasks in NLP field. So far, several NLP tools have been open source, including NLTK [46], StanfordNLP [47], SpaCy. Among them, SpaCy is a fast, powerful and lightweight NLP tool for various languages, of which the functions include tokenizer, tagger, parser, etc. In addition, SpaCy achieves a precision of 98% on part of speech tagging, and 95% on parsing, which can be considered as a reliable NLP tool.

Syntactic Features should be one of the important features for NER. Hamon et al. [48] constructed rules based on syntactic features and implemented BioNER based on these rules. However, at present, the mainstream methods mainly consider the contextual features of sentences and implement NER based on neural networks. And there are few NER methods paying attention to both contextual and syntactic features. [32] uses a key-value memory network to fuse syntactic information, which ignores the topological information of sentences to some extent.

### Graph neural networks

Recently, considering that data in some application scenarios are generated from non-Euclidean spaces, researchers pay more attention to applying deep learning technology to graphic data, and the graph neural network (GNN) comes into being [49].

As one of the GNNs, GCN [50] analyzes the feature of a node based on both the node and its neighbors and can implement tasks such as node classification, link prediction, and recommendation. However, GCN assigns the same weight to neighbor nodes, and GCN fails to fuse node features in dynamic graphs. Different from the fixed kernel of GCN, GAT [14] is a graph neural network based on the masked self-attention mechanism which dynamically calculates weights for neighbors according to the topology of a graph and further generates the representation of the central node.

GNNs have also been applied in NER task recently. [51] uses BERT and 2 GNN layers to implement NER in general domain. As for syntactic features in their work, only dependency graph is used, which underutilizes the part of speeches and other dependency features [52]. uses BERT and a GCN layer to implement a nested NER task. They first tag part of speeches and dependencies based the proposed model, and optimize the word representation through a GCN layer. However, the GCN used in their work is not suitable for various and flexible structure of sentences, which may cause a certain limitation.

## Methodology

### Formulating the BioNER task as a node classification problem

To introduce the syntactic features, we use an undirected graph to model the topology of a sentence. Robinson pointed out that only one element is independent in a sentence, and all others depend directly on some element [53]. In other words, the dependencies of a sentence can be modeled via a graph, where elements/tokens are modeled as nodes, and dependencies are modeled as edges between nodes. In addition, features of elements/tokens, including distributed representations, are modeled as attributions of the corresponding nodes. An example of using the graph to model the topology of a sentence is shown in Fig. 1.

SpaCy (version 3.2.1, with the package en_core_web_trf) is utilized in this paper to parse sentences, and for a sentence ${s}_{i}$, part of speeches ${P}_{i}=({p}_{1}, {p}_{1}, \dots , {p}_{n})$, and dependencies ${Dep}_{i}=\left\{\left({h}_{1},{t}_{1},{d}_{1}\right), \left({h}_{2},{t}_{2},{d}_{2}\right), \dots , \left({h}_{m},{t}_{m},{d}_{m}\right)\right\},$ are thereout obtained, where ${p}_{j}$ is the corresponding part of speech of the $j$-th token, and $\left({h}_{l},{t}_{l},{d}_{l}\right)$ represents the head token, tail token, and type of dependency in the $l$-th dependency triple. Then, the corresponding nodes of ${h}_{l}$ and ${t}_{l}$ are neighbors of each other in the graph. Moreover, part of speeches and dependencies are also further encoded, which is presented in Section C.

We also construct a $Adjacent\_Matrix$ to quantize the topology of a sentence, which is a symmetric matrix. For ${a}_{ij}\in Adjacent\_Matrix$ we have,

1 $$a_{ij} = \left\{ {\begin{array}{*{20}l} {1,} \hfill & {if\;i\;is\;j^{\prime}s\;neighbor} \hfill \\ {0,} \hfill & {else} \hfill \\ \end{array} } \right.$$

After converting sentences of datasets into a set of graphs, the BioNER task can then be formulated as a node classification problem. Each graph is composed of nodes with attributes and edges, where a node represents a token, an edge represents the dependency between tokens and an attribute indicates the distributed representation of the corresponding token. And our goal is to classify the above nodes into corresponding labels.

### Overall architecture of BioByGANS

**Fig. 2:**
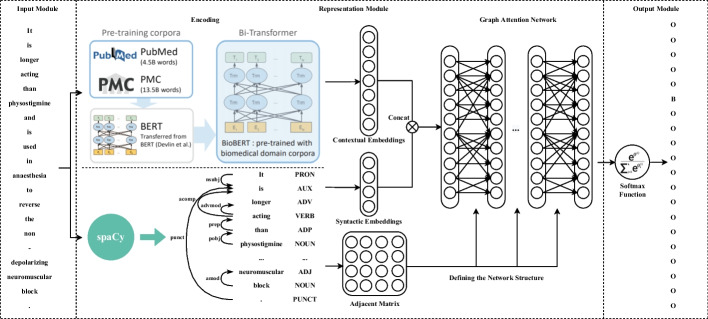
The overall architecture of BioByGANS. Sentences are input into BioBERT to get contextual embeddings. Meanwhile, SpaCy is used to parse sentences and get syntactic embeddings according to part of speeches and dependencies, and adjacent matrix based on dependencies. A graph attention network layer, which is defined based on the adjacent matrix, receives the concatenation of contextual and syntactic embeddings as the input, and generates a fusing node representation considering both the contextual features and syntactic features. Last, a softmax function is used to calculate the probabilities and get the results as BIO labels

We propose an end-to-end model for BioNER in the node classification framework. The proposed model, BioByGANS, is ulteriorly divided into 3 modules, including the input, representation, and output module. The overall architecture is shown in Fig. 2.

First, for biomedical texts, we use periods to segment sentences and spaces and symbols to segment words. And thus, each sentence is transformed into a sequence of tokens. Second, contextual features and syntactic features of a sentence are encoded via the BioBERT and SpaCy respectively. In addition, the topology of a sentence is converted into an adjacent matrix through SpaCy. A graph attention network is then used to generate representations of nodes based on the adjacent matrix. The mutli-head attention mechanism is also utilized to capture richer information in sentence graphs. Last, we use a softmax function to compute the probability distribution of labels using the final distributed representation of a node, and get the output.

### Encoding module of BioByGANS

In this paper, we use BioBERT [11] and SpaCy [13] to encode contextual and syntactic features respectively. The outputs,$Cont$ and $Synt$, are 768-dimensional and 54-dimensional vectors respectively for each token.

2 $$Cont=BioBERT\left(Sentence\right) Synt=SpaCy\left(Sentence\right)$$

Specifically, syntactic features are divided into part of speeches and dependencies. We first get the statistics about the frequency distribution of part of speeches and dependencies of biomedical named entities in corpora, and we use a frequency of 1% as the threshold to filter part of speeches and dependencies. Then, we get 10 outstanding part of speeches, including ‘NOUN’, ‘PROPN’, ‘PUNCT’, ‘NUM’, ‘ADJ’, ‘VERB’, ‘SYM’, ‘CCONJ’, ‘ADP’, and ‘PART’, and 18 outstanding dependencies, including ‘compound’, ‘punct’, ‘nmod’, ‘amod’, ‘pobj’, ‘conj’, ‘appos’, ‘det’, ‘nummod’, ‘npadvmod’, ‘cc’, ‘nsubj’, ‘dobj’, ‘prep’, ‘nsubjpass’, ‘acl’, ‘root’ and ‘case’.

**Fig. 3:**
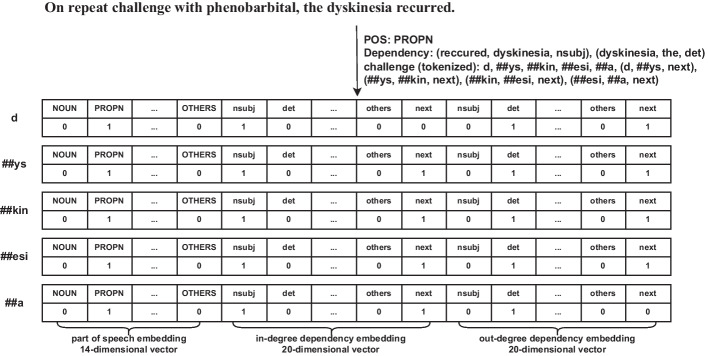
An example of using the one-hot strategy to encode syntactic features. In this example, dyskinesia, which is a named entity, is a “PROPN” in the sentence, and has 2 dependencies with other words. Moreover, the tokenizer of BioBERT cuts dyskinesia into 5 word-pieces, which inherit the syntactic features of the original word in the sentence, and are linked by “next” to indicate the order of word-pieces

In addition, for part of speeches, we introduce ‘OTHERS’ to represent the set of indistinctive part of speeches. In addition, we also introduce ‘CLS’, ‘SEP’, and ‘X’ for BioBERT’s tokenization, where ‘CLS’ represents the start tag of a sentence, ‘SEP’ represents the end tag of a sentence, and ‘X’ represents tokens starting with ‘##’. For dependencies, we introduce ‘others’ to represent the set of indistinctive dependencies, and we introduce ‘next’ to link the tokens which are segmented from a single word. Moreover, given that dependencies have directions, we make a distinction between in-degree and out-degree for each dependency, which means each dependency is represented by two dimensions. We choose the one-hot strategy to encode the above features instead of trainable parameters which is randomly initialized to learn co-relations between syntactic labels. Because the unremarkable part of speeches and dependencies are aggregated as ‘OTHERS’ and ‘others’ respectively, and the learning process of co-relations between redefined labels may introduce additional confusion. And we get a vector of 54 dimensions for a token, which is shown in Fig. 3.

### Graph attention network module of BioByGANS

In this paper, based on the result of the encoding module, we use a GAT to generate the distributed representation, $Rep$, that is,

3 $$Rep=GAT(Cont|\left|Synt\right)$$

where $||$ denotes concatenation operation. The $Rep$ is modeled as the attribute of each node, which is utilized for classification.

Graph Attention Network (GAT) [14] is a graph neural network based on the masked self-attention mechanism. and can dynamically calculate weights for neighbors according to the topology of a graph and further generates the representation of the central node. And in this paper, we design a GAT for sentence graphs.

First, we use a linear transformation to calculate the coefficient between node $i$ and node $j$. That is,

4 $${coef}_{ij}=LeakyReLU({\overrightarrow{\mathbf{a}}}^{T}[\mathbf{W}{\overrightarrow{h}}_{i}||\mathbf{W}{\overrightarrow{h}}_{j}]) , j\in {N}_{i}$$

where ${\overrightarrow{h}}_{i}$ and ${\overrightarrow{h}}_{j}$ donates the initial attributes of node $i$ and $j$ respectively, ${N}_{i}$ donates the set of neighbors of node $i$, $\mathbf{W}\in {{\varvec{R}}}^{F\times {F}^{\mathrm{^{\prime}}}}$ and $\overrightarrow{{\varvec{a}}}\in {{\varvec{R}}}^{{2F}^{\mathrm{^{\prime}}}\times 1}$ are trainable matrices, $||$ denotes concatenation operation, and $T$ denotes transposition operation.

Next, we normalize the coefficients using a softmax function,

5 $${\alpha }_{ij}={softmax}_{j}\left({coef}_{ij}\right)=\frac{\mathrm{exp}\left({coef}_{ij}\right)}{{\sum }_{k\in {N}_{i}}\mathrm{exp}\left({coef}_{ik}\right)}$$

Then, the normalized attention coefficients are used to calculate the attribute of the central node $i$,

6 $${\overrightarrow{h}}_{i}^{\mathrm{^{\prime}}}=\sigma \left({\sum }_{j\in {N}_{i}}{\alpha }_{ij} \mathbf{W}{\overrightarrow{h}}_{j}\right)$$

where $\sigma$ is an activation function, and we use an ELU function in this paper.

We also introduce a multi-head attention mechanism to enhance the ability of our model. Each independent attention head calculates coefficients according to Eq. 5 and generates a representation of the central node according to Eq. 6. And the outputs of attention heads are concatenated.

7 $${\overrightarrow{\mathrm{h}}}_{i}^{\mathrm{^{\prime}}}={||}_{m=1}^{M} \sigma \left({\sum }_{j\in {N}_{i}}{\alpha }_{ij}^{m} {\mathbf{W}}_{m}{\overrightarrow{h}}_{j}\right)$$

where $||$ denotes concatenation operation, ${\alpha }_{ij}^{m}$ donates the coefficient from the $m$ th attention head and ${\mathbf{W}}_{m}\in {{\varvec{R}}}^{{F}^{\mathrm{^{\prime}}}}$ donates the transformation matrix of the $m$ th attention head. In this way, the initial attributes $({\overrightarrow{h}}_{1}, {\overrightarrow{h}}_{2}, \dots , {\overrightarrow{h}}_{n})$ are transferred into final attributes $({\overrightarrow{h}}_{1}^{\mathrm{^{\prime}}}, {\overrightarrow{h}}_{2}^{\mathrm{^{\prime}}}, \dots , {\overrightarrow{h}}_{n}^{\mathrm{^{\prime}}})$ through a GAT, which is used to make the final inference.

## Results and discussion

### Datasets and experimental settings

We develop the project in the TensorFlow environment. We use 8 biomedical corpora covering genes, proteins, species, diseases, and chemicals. The statistics of the datasets are presented in Table 1, and a description of the datasets is as follows.

- *BC2GM* [54] is a dataset for the BioCreative II Gene Mention Recognition task. It is composed of 20130 sentences from abstracts of biomedical publications and is annotated with more than 24000 gene and protein mentions.
- *JNLPBA* [55] is a biomedical dataset derived from the GENIA version 3.02 corpus and created by a controlled search on MEDLINE. It includes over 2000 abstracts of biomedical publications and is annotated with multiple classes of entity types.
- *Species-800* [56] is a manually annotated corpus, which is composed of 800 publications from PubMed. And entities mainly belong to the species category.
- *LINNAEUS* [57] is an open-source corpus for species entities and is composed of annotated full-text data from 100 publications. Additionally, entities in LINNAEUS are also normalized and mapped to NCBI taxonomy IDs. And it is also used by BioNER tools such as BioBERT as a benchmark.
- *BC5CDR* [58] is a dataset for BioCreative V Chemical-Disease Relation Recognition task. For the BioNER task, it can also be divided into two categories, BC5CDR-Disease and BC5CDR-Chemical. It is composed of the abstracts of biomedical publications and annotated with chemicals, disease, and chemical-disease relationships in texts.
- *NCBI-Disease* [59] is composed of abstracts of 793 publications in PubMed and annotated with nearly 7000 disease mentions. In addition, most of the mentions are mapped to the NCBI concept vocabulary.
- *BC4CHEMD* [60] is a dataset for BioCreative IV Chemical Compound and Drug Name Recognition task. It is an open-source, manually annotated chemical corpus, and consists of 10000 abstracts of PubMed publications.

**Table 1:** Statistics of the biomedical datasets

Entity type	Corpus name	Annotated sentences	Sentence max length
Gene/Protein	BC2GM	20,130	206
	JNLPBA	22,401	221
Species	Species-800	8193	143
	LINNAEUS	23,151	246
Disease	BC5CDR-Disease	13,938	225
	NCBI-Disease	7287	123
Chemical	BC4CHEMD	87,674	225
	BC5CDR-Chemical	13,938	225

Datasets we use in this paper are pre-preprocessed and provided by Lee et al. [11], each of which is divided into training, developing, and testing sets, and we use precision, recall, and F1-score to evaluate our model, of which the calculation formulas are as follows.

8 $$Precision=\frac{TP}{TP+FP}$$

9 $$Recall=\frac{TP}{TP+FN}$$

10 $$F1\_Score=\frac{2\times Precision\times Recall}{Precision+Recall}$$

where TP donates true positive, FP donates false positive, and FN donates false negative. Specifically, during the inferring process, a positive of our model contains all the words in its left and right boundaries (entities with multi-word must be completely captured), which is the same strategy of the baseline methods in the following section. Moreover, hyper-parameters of BioByGANS are listed in Table 2, where msl denotes max sequence length, bs means batch size, lr means learning rate of the model, and layer means the number of GAT layers, and head and unit mean the numbers of heads and units in each GAT layer.

**Table 2:** The hyper-parameters of BioByGANS

Dataset	msl	bs	lr	Layer	Head	Unit
BC2GM	256	32	5e−5	2	12	64
BC4CHEMD	256	32	3e−5	4	12	64
BC5CDR-chem	256	32	3e−5	4	12	64
BC5CDR-disease	256	32	3e−5	4	12	64
NCBI-disease	256	32	5e−5	4	8	96
JNLPBA	256	32	5e−5	2	8	96
LINNAEUS	256	32	5e−5	4	12	64
Species-800	256	32	5e−5	2	12	64

### BioByGANS vs. baseline methods on performance

In this section, the baseline models used in comparison to our proposed model are presented, and the results of baseline models are obtained from their original publications. Some of the following methods focuses on one or more biomedical corpora, and only BioBERT has results for all 8 corpora.

Tables 3, 4, 5 and 6 shows the experimental results on BioNER datasets, where p denotes precision, r denotes recall, and f1 denotes f1-score. We choose thChem [25], TaggerOne [27], BiLSTM-CRF [28], Att-BiLSTM-CRF [20], CollaboNet [29], DABLC [30], MTM-CW [31], BioKMNER [32], BioELECTRA [35], BioBERT-MRC [33], MTL-LS [34], and BioBERT [11] as our baseline methods. And BioBERT is the main baseline for our model.

**Table 3:** Comparison of BioNER for chemicals

Method\Dataset	BC4CHEMD			BC5CDR-chemical		
	p	r	f1	p	r	f1
tmChem [25]	89.09	85.75	87.39	–	–	–
TaggerOne [27]	–	–	–	94.20	88.80	91.40
BiLSTM-CRF [28]	91.31	87.73	89.48	92.82	88.52	90.62
Att-BiLSTM-CRF [20]	92.29	90.01	91.14	93.49	91.68	92.57
CollaboNet [29]	–	–	–	94.26	92.38	93.31
MTM-CW [31]	91.30	87.53	89.37	–	–	–
BioKMNER [32]	–	–	–	–	–	94.00
BioBERT-MRC [33]	**93.89**	91.96	92.92	94.37	94.00	94.19
MTL-LS [34]	–	–	92.42	–	–	93.83
BioELECTRA[35]	–	–	–	–	–	93.60
BioBERT [11]	92.80	91.92	92.36	93.68	93.26	93.47
**Proposed**	93.42	**92.52**	**92.97**	**94.53**	**94.95**	**94.74**

Bold indicates the best performances of models in each subtask

**Table 4:** Comparison of BioNER for diseases

Method\Dataset	NCBI-disease			BC5CDR-disease		
	p	r	f1	p	r	f1
TaggerOne [27]	85.10	80.80	82.90	85.20	80.20	82.60
BiLSTM-CRF [28]	86.11	85.49	85.80	87.60	86.25	86.92
CollaboNet [29]	85.61	82.61	84.08	85.61	82.61	84.08
MTM-CW [31]	85.86	86.42	86.14	**89.10**	88.47	**88.78**
DABLC [30]	88.30	89.01	88.60	**89.10**	87.50	88.30
BioKMNER [32]	–	–	90.08	–	–	–
BioBERT-MRC [33]	89.67	90.42	90.04	88.61	87.07	87.83
MTL-LS [34]	–	–	89.25	–	–	87.28
BioELECTRA[35]	–	–	89.38	–	–	85.84
BioBERT [11]	88.22	91.25	89.71	86.47	87.84	87.15
**Proposed**	**89.99**	**93.20**	**91.57**	86.69	**88.82**	87.74

Bold indicates the best performances of models in each subtask

**Table 5:** Comparison of BioNER for genes and proteins

Method\dataset	BC2GM			JNLPBA		
	p	r	f1	p	r	f1
BiLSTM-CRF [28]	81.57	79.48	80.51	71.35	75.74	73.48
CollaboNet [29]	80.49	78.99	79.73	74.43	83.22	78.58
MTM-CW [31]	82.10	79.42	80.74	70.91	76.34	73.52
BioKMNER [32]	-	-	84.92	-	-	77.72
BioBERT-MRC [33]	**87.04**	83.98	**85.48**	**75.96**	82.13	78.93
MTL-LS [34]	**–**	**–**	82.92	**–**	**–**	**–**
BioELECTRA[35]	**–**	**–**	84.69	**–**	**–**	**80.07**
BioBERT [11]	84.32	85.12	84.72	72.24	83.56	77.49
**Proposed**	84.97	**85.32**	85.15	72.69	**84.54**	78.16

Bold indicates the best performances of models in each subtask

**Table 6:** Comparison of BioNER for species

Method\dataset	LINNAEUS			Species-800		
	p	r	f1	p	r	f1
BioKMNER [32]	–	–	88.79	–	–	**76.21**
MTL-LS [34]	–	–	86.37	–	–	–
BioBERT [11]	90.77	85.83	88.24	**72.80**	75.36	74.06
**Proposed**	**93.91**	**88.25**	**90.99**	71.53	**78.83**	75.01

Bold indicates the best performances of models in each subtask

Compared with other methods, the proposed model and BioBERT get good performance in most datasets, which indicates models based on pre-training strategy are more stable than other methods. Compared with BioBERT, our model gets significant promote on recall in all 8 datasets, which increases 3.47% at most (Species-800), due to the supplement of syntactic features. That is, if a token, of which the part of speech is ‘NOUN’, is linked with a token with ‘VERB’ as its part of speech through ‘nsubj’, the former has a higher probability of being an entity. In addition, our model gets promote on precision in 7 datasets, which increases 1.10% in average,except Species-800 (−1.27%). Overall, the proposed model gets higher F1-score in all 8 datasets than BioBERT, which increases 2.75% at most and 0.43% at least.

As shown in Tables 3, 4 and 5, BioBERT-MRC gets a slimly better precision in BC4CHEMD, and gets better precision in BC5CDR-disease, BC2GM and JNLPBA than our model because its queries are pre-defined based on prior knowledge in biomedical field and suitable for the above datasets. However, BioBERT-MRC gets both worse precision in NCBI-Disease and BC5CDR-Chemical and worse recall in all datasets than our model, which indicates the performance of BioBERT-MRC depends on the quality of the queries and matched-degree between queries and corpora. In addition, a prerequisite of BioBERT-MRC is carefully designed queries, which needs more manual costs and means additional prior information beyond the original corpus is transmitted to the model. Compared to the automatically end-to-end processing of the same corpus of our model, the result of BioBERT-MRC is biased to some extent, and the performance of BioBERT-MRC is not robust enough.

As shown in Tables 4 and 5, MTM-CW gets better precision and F1 score in BC5CDR-Disease, CollaboNet gets a better precision and BioELECTRA gets a better F1-score in JNLPBA than our model. However, the above 3 methods get poorer precision, recall, and F1-score than the proposed model in other datasets, which indicates their performance is unstable to some extent. For instance, MTM-CW gets 4.64% lower on F1-score (JNLPBA), BioELECTRA gets 1.90% lower on F1-score (BC5CDR-Disease) and CollaboNet gets 4% lower on all evaluation metrics (BC2GM) than our model. CollaboNet and MTM-CW are multi-task models, and the above 2 corpora have both annotations for multiple entity types in each sentence, which means multi-task learning may improves the model for corpora with annotations of multi-type entities.

As shown in Table 6, although BioKMNER gets better F1-score in Species-800, it gets poorer performances in any other datasets than our model. For example, BioKMNER achieves 1.49% lower on F1-score (NCBI-disease) than our model.

Overall, our model outperforms other models in BioNER tasks for different entities in most datasets on precision, recall and F1 score because of its ability to capture both contextual and syntactic features, and make good use of the topology information.

### The effect of different parameters of GAT on performance

In this section, we first compare the effect of various layers of GAT on performance of BioByGANS. Specifically, we fix the number of heads and units of each layer as 12 and 64, and prepare 5 alternative parameters, 1, 2, 4, 8, and 12, to explore the effect of GAT layer change. In addition, we use 4 corpora, BC2GM, BC5CDR-chem, BC5CDR-disease, and Species-800, for the test.

**Fig. 4:**
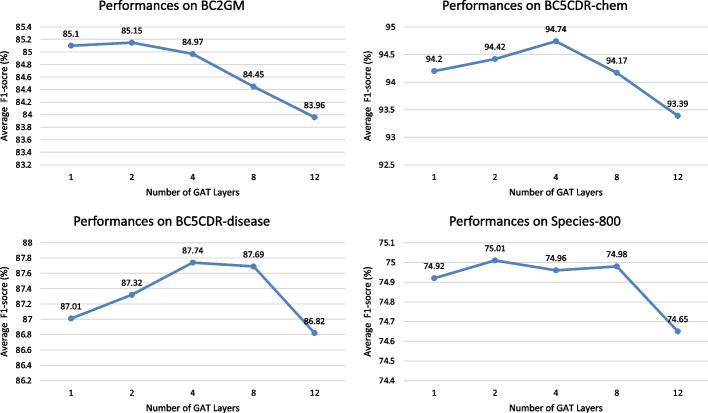
The performance comparison for different number of GAT layers

Figure 4 illustrates the performance comparison for various layers of GAT. This experiment result shows that appropriately increasing layers of GAT are useful for BioNER tasks on most corpora. Moreover, we noticed that the performance on BC5CDR-chem with 4 GAT layers (94.74% in the average F1-score) is superior to it with 1 GAT layer (94.20% in the average F1-score). On the other hand, if the GAT is too deep, the model may get the poor performance. The performance on BC5CDR-chem with 4 GAT layers is superior to it with 12 GAT layer (93.39% in the average F1-score), which is caused by overfitting. As the result shows, 2 or 4 is a suitable choice for the number of GAT layers. In addition, as shown in Fig. 4, our model gets best performance on BC2GM and Species-800 with 2 GAT layers, while on BC5CDR-chem and BC5CDR-disease with 4 GAT layers. We investigate sentences from these biomedical datasets and notice that the characteristics of the syntactic expressions are quite different from each other because research papers on different topics may have their own sentence structures and style, which indicates that the choice of numbers of GAT layer depends on the syntactic expression of sentences in corpora.

Then, we compare the effect of various heads&units of each GAT layer on performance. To guarantee the output dimension fixed, we make the product of units and heads in each layer constant. Specifically, we fix the number of layers as 1, and prepare 6 alternative parameter groups, (1,768), (2,384), (4,192), (6,128), (8,96), and (12,64), to explore the effect of GAT head&unit change. In addition, we use 2 corpora, BC5CDR-chem and NCBI-disease for the test.

**Fig. 5:**
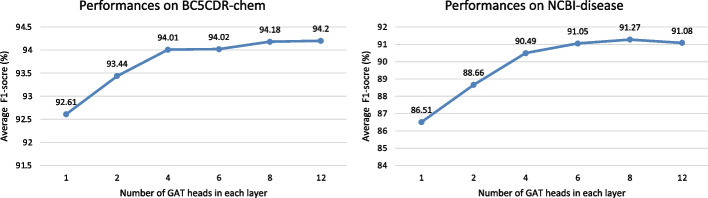
The performance comparison for different number of head&units for each GAT layer

Figure 5 illustrates the performance comparison for various heads&units of GAT. This experiment result shows the GAT with multi-head attention mechanism obtains a more comprehensive representation for nodes. We noticed that the model gets the best performances with 12 heads on BC5CDR-chem, and 8 heads on NCBI-disease, which is better than the model without additional attention heads. Overall, the multi-head attention mechanism can get node features from different aspects, and the parameters of attention heads and units need to be selected according to the actual data.

### Ablation studies

To better understand the relative importance of each component for the token representation, we perform ablation studies in this section. Specifically, we fix the number of heads and units of each layer as 12 and 64, and layers as 4. Moreover, we use BC5CDR-chem for the test. As shown in Table 7, removing the syntactic features significantly impairs the performance of the model, of which F1-score drops 1.24%.

**Table 7:** Ablation studies on BC5CDR-chem corpus

Representation component	Precision	Recall	F1-score
BioBERT + POS + Dependency	**94.53**	**94.95**	**94.74**
-POS	94.11	94.41	94.26
-Dependency	94.10	94.74	94.42
-POS and Dependency	93.13	93.87	93.50

Bold indicates the best performances of models in each subtask

Then, we evaluate the impact of part of speech (POS) and dependency solely. And the results show that model with part of speech component increases 0.92% in F1-score, and model with dependency increases 0.76% in F1-score, which means the syntactic features do help improve the model performance. Moreover, according to the present results, it can be concluded that both POS and dependency contribute to model performance. We also notice that model without dependency performs better than model without POS, which may indicate that POS is a more important feature for NER, or that a portion of dependency feature (geometric topology) has been utilized in the form of adjacent matrix in GAT layers. However, the syntactic parse results from SpaCy are not 100% precise. Especially, the performance of SpaCy for POS tagging is better than that for dependency parsing. Therefore, the above result is also probably because SpaCy leads more noise in dependency parsing than POS tagging. Above all, it is difficult to assert which contributes more to the NER task, POS or dependency. Further research is needed to prove which is more important to NER task.

Moreover, we investigate the impact of the topological feature to our model. A model with dependency represents a model with topological information, which is because topology contains not only the graph structure (geometry), but also attributes of nodes and edges in graph, which are both from the syntactic dependency tree. As shown in Table 7, model without POS (“-POS”) outperforms model without POS and dependency (“-POS & Dependency”), and gets a promotion of 0.98%, 0.54% and 0.76% on precision, recall and f1-score respectively, which indicates the ability of our model to capture topological information, and topology does have positive effects on model performance.

## Discussion and limitation

Our model achieves better performances for the following reasons. (1) BioByGANS learns better word representations from BioBERT and SpaCy respectively, which involves contextual and syntactic features. (2) We use a graph to model the topology of a sentence and formulate the BioNER task as a node classification problem, which is then solved through GAT layers. Because the topology of a sentence is more of a graph than a sequence according to the constituency and dependency.

As for the generalization of our model, the proposed model framework is able to be migrated to other domains instead of being limited to the biomedical domain. BioNER is more complicated than NER in general domains to some extent. Compared with general texts, biological texts contain more entity types, and each sentence may contain multiple types and numbers of named entities. Besides, biological texts contain longer and more complex sentences. Hence, the proposed model may also get good performances in NER of other fields theoretically.

As for the processing time of the model, we have conducted experiments to test the speed of our model in training process and inferring process. The experimental environment is a 24-core, Inter® Xeon® Gold 6248R CPU, 3.0 GHz-frequency, with a single A100 PCIE 40 GB GPU and 512 MB-memory server. The operating system is 64-bit Ubuntu 16.04.4 LTS (GNU / Linux 4.13.0–36 -generic x86_64). And we have performed the test on BC5CDR-chem dataset, with epochs as 50, max sequence length as 256, batch size as 32, learning rate as 3e-5, graph attention layers as 4, and head&units as 12&64. As for the training process, the model cost about 95 min, where it took about 5 min to preprocess the sentences in training set of BC5CDR-chem, and it took about 90 min to train the neural network architecture, including fine-tuning BioBERT and training the graph attention network and classifier. And as for the inferring process, our model implemented NER for more than 1700 sentences in a minute, which indicates the efficiency of our proposed model. One point that should be concerned about the inferring process is that the calculation of the neural network is fast, which took only about 50 s, while the preprocessing time for sentences in testing set was about 2 min. Considering the CPU-based preprocessing is independent from the GPU-based neural network inferring, our model can be further accelerated through pipelining and parallel processing. We have also tested the speed of BioBERT with the same hyper-parameters, of which the result is BioBERT took about 60 min to training and fine-tuning, and it could process sentences in testing set of BC5CDR-chem in about 35 s during the inferring process. Considering the significant improvement in performance compared with BioBERT, the extra time cost of our model is tolerable. In addition, [51] and [52] also applied SpaCy to preprocess the syntactic features, which also indicates SpaCy is a reliable and efficient tool to implement this task and saves as much processing time as possible.

However, the proposed model has some limitations. First, errors from SpaCy in tagging part of speeches and dependencies may cause the error drift of our model. For example, using a NLP tool which is specifically for biomedical field may reduce the error drift to some extent [61]. The transformer-based biomedical language package for SpaCy need to be trained in the future work. In addition, a transfer learning strategy which combines BioNER with the tagging of syntactic labels may also help to improve the model. Second, as for the decoder of BioByGANS, the softmax function fails to take advantage of the transition probability of labels between nodes, which may cause the error of our model. As can be seen in Table 4, precision on BC5CDR-disease of our model is not good enough, so we investigate the error instances, which is shown in Table 8. For case 1&2, a coordinative component of disease entities and an object structure which describe pathological states are recognized respectively. For case 3, the modifier of an entity is recognized as the beginning of it. That means, the model expands the searching scope of entities according to syntactic features, which increases the recall but sacrifices the precision.

**Table 8:** Error instances in BC5CDR-disease dataset

No	Gold standard	Result of BioByGANS
1	**Azotemia**, body fluid insufficiency and **bacterial infections** were frequently found in these patients	**Azotemia**, **body fluid insufficiency** and **bacterial infections** were frequently found in these patients
2	RESULTS: Both forms of stress led to prolonged but reversible systolic and diastolic dysfunction	RESULTS: Both forms of stress led to prolonged but reversible **systolic and diastolic dysfunction**
3	A case of bilateral **optic neuropathy** in a patient on tacrolimus (FK506) therapy after liver transplantation	A case of **bilateral optic neuropathy** in a patient on tacrolimus (FK506) therapy after liver transplantation

Bold indicates the best performances of models in each subtask

## Conclusion

In this paper, we use BioBERT in the node classification framework to implement BioNER and propose an end-to-end method, BioByGANS. Compared with using BioBERT in the sequence labeling framework, BioByGANS has a significantly stronger ability to recognize various biomedical entities. Moreover, the proposed model can solve the problem of underutilizing syntactic features. The experiment results show that our model outperforms different baseline models in most biomedical datasets, which demonstrates the effectiveness and robustness of BioByGANS. In the future, we plan to design a decoder for the graphic topology, such as a nonlinear-chain CRF, to get better performances for distributed representations generated by graph neural networks in the node classification framework. Moreover, we plan to search for a better syntactic parsing tool which is specifically for biomedical texts and based on deep neural networks to achieve better performance. Multi-task learning should also be considered to improve the model.

## Data Availability

We make the source code and model available at https://github.com/zxw1995shawn/BioByGANS.

## Abbreviations

BioNER: Biomedical named entity recognition
BioBERT: Biomedical bidirectional encoder representations from transformers
NER: Named entity recognition
OOV: Out of vocabulary
NLP: Natural language processing
LSTM: Long short Term Memory
CRF: Conditional random field
SOTA: State of the art
GAT: Graph attention network
CBOW: Continuous bag of words
MLM: Masked language model
NSP: Next sentence prediction
GNN: Graph neural network
GCN: Graph convolutional network

## References

[1] Chinchor N, Robinson P (1997). MUC-7 named entity task definition. Proc 7th Conf Message Underst.

[2] Alshaikhdeeb B, Ahmad K (2016). Biomedical named entity recognition: a review. Int J Adv Sci Eng Inf Technol.

[3] Perera N (2020). Named entity recognition and relation detection for biomedical information extraction. Front Cell Dev Biol.

[4] LeCun Y (2015). Deep learning. Nature.

[5] Sutskever I (2014). Sequence to sequence learning with neural networks. Proc 27th Int Conf Neural Inf Process Syst.

[6] Mikolov T et al. Efficient estimation of word representations in vector space. In: Proceedings of Workshop at International Conference on Learning Representations (ICLR). 2013.

[7] Peters ME et al. Deep contextualized word representations. In: Proceedings of the 2018 Conference of the North American Chapter of the Association for Computational Linguistics: Human Language Technologies, New Orleans, Louisiana. 2018; 1, p. 2227–2237

[8] Devlin J et al. Bert: pre-training of deep bidirectional transformers for language understanding. In: Proceedings of the 2019 Conference of the North American Chapter of the Association for Computational Linguistics: Human Language Technologies. 2018; 1, p. 4171–4186.

[9] Zhang Y (2019). BioWordVec, improving biomedical word embeddings with subword information and MeSH. Sci Data.

[10] Jin Q et al. Probing biomedical embeddings from language models. In: Proceedings of the 3rd Workshop on Evaluating Vector Space Representations for NLP. 2019; p. 82–89.

[11] Lee J (2020). BioBERT: a pre-trained biomedical language representation model for biomedical text mining. Bioinformatics.

[12] Fromkin V (2013). An Introduction to Language.

[13] Honnibal M, Montani I. spaCy 2: Natural language understanding with Bloom embeddings, convolutional neural networks and incremental parsing. 2017; Homepage: https://spacy.io/.

[14] Veličković, P., et al. Graph attention networks. In: Proceedings of International Conference on Learning Representations (ICLR). 2018.

[15] Fukuda K-I (1998). Toward information extraction: identifying protein names from biological papers. Pac Symp Biocomput.

[16] Krauthammer M (2000). Using BLAST for identifying gene and protein names in journal articles. Gene.

[17] Kazama JI et al. Tuning support vector machines for biomedical named entity recognition. In: Proceedings of the ACL-02 workshop on Natural language processing in the biomedical domain, PA, USA. 2002; 3, p. 1–8.

[18] Zhao S. Named entity recognition in biomedical texts using an HMM model. In: Proceedings of the International Joint Workshop on Natural Language Processing in Biomedicine and its Applications (NLPBA/BioNLP), 2004; p. 87–90.

[19] Song B (2021). Deep learning methods for biomedical named entity recognition: a survey and qualitative comparison. Brief Bioinform.

[20] Luo L (2018). An attention-based BiLSTM-CRF approach to document-level chemical named entity recognition. Bioinformatics.

[21] Dang TH (2018). D3NER: biomedical named entity recognition using CRF-biLSTM improved with fine-tuned embeddings of various linguistic information. Bioinformatics.

[22] Tong F et al. A deep network based integrated model for disease named entity recognition. In: Proceedings of 2017 IEEE International Conference on Bioinformatics and Biomedicine (BIBM), 2017; p. 618–621.

[23] Tong F et al. Using deep neural network to recognize mutation entities in biomedical literature. In: Proceedings of 2018 IEEE International Conference on Bioinformatics and Biomedicine (BIBM), 2018; p. 2329–2332.

[24] Wei C-H (2013). tmVar: a text mining approach for extracting sequence variants in biomedical literature. Bioinformatics.

[25] Leaman R (2015). tmChem: a high performance approach for chemical named entity recognition and normalization. J Cheminform.

[26] Wei C-H (2015). GNormPlus: an integrative approach for tagging genes, gene families, and protein domains. BioMed Res Int.

[27] Leaman R, Lu Z (2016). TaggerOne: joint named entity recognition and normalization with semi-Markov Models. Bioinformatics.

[28] Lample G et al. Neural architectures for named entity recognition. In: Proceedings of the 2016 Conference of the North American Chapter of the Association for Computational Linguistics: Human Language Technologies, 2016; p. 260–270.

[29] Yoon W (2019). Collabonet: collaboration of deep neural networks for biomedical named entity recognition. BMC Bioinform.

[30] Xu K (2019). Document-level attention-based BiLSTM-CRF incorporating disease dictionary for disease named entity recognition. Comput Biol Med.

[31] Wang X (2019). Cross-type biomedical named entity recognition with deep multi-task learning. Bioinformatics.

[32] Tian Y (2020). Improving biomedical named entity recognition with syntactic information. BMC Bioinform.

[33] Sun C (2021). Biomedical named entity recognition using BERT in the machine reading comprehension framework. J Biomed Inform.

[34] Chai Z (2022). Hierarchical shared transfer learning for biomedical named entity recognition. BMC Bioinform.

[35] Kanakarajan K et al. BioELECTRA: pretrained biomedical text encoder using discriminators. In: Proceedings of the 20th Workshop on Biomedical Language Processing. 2021; p. 143–154.

[36] Bellegarda JR (2004). Statistical language model adaptation: review and perspectives. Speech Commun.

[37] Mikolov T et al. Distributed representations of words and phrases and their compositionality. In: Proceedings of the 26th International Conference on Neural Information Processing Systems-Volume 2, NY, USA. 2013; 2, p. 3111–3119.

[38] Zhang Y (2010). Understanding bag-of-words model: a statistical framework. Int J Mach Learn Cybern.

[39] Ramos J (2003). Using tf-idf to determine word relevance in document queries. Proc First Instr Conf Mach Learn.

[40] Joulin A et al. Fasttext. zip: Compressing text classification models. arXiv preprint arXiv:1612.03651; 2016.

[41] Pennington J et al. Glove: Global vectors for word representation. In: Proceedings of the 2014 conference on empirical methods in natural language processing (EMNLP), 2014; p. 1532–1543.

[42] McCann B et al. Learned in translation: Contextualized word vectors. In: Proceedings of the 31st International Conference on Neural Information Processing Systems, NY, USA, 2017; p. 6297–6308.

[43] Radford A et al. Improving language understanding by generative pre-training. 2018.

[44] Hochreiter S, Schmidhuber J (1997). Long short-term memory. Neural Comput.

[45] Vaswani A et al. Attention is all you need. In: Proceedings of the 31st International Conference on Neural Information Processing Systems, 2017; p. 6000–6010.

[46] Bird S, Loper E. NLTK: the natural language toolkit. In: Proceedings of the ACL-02 Workshop on Effective tools and methodologies for teaching natural language processing and computational linguistics. 2004; 1, p. 63–70.

[47] Manning CD et al. The Stanford CoreNLP natural language processing toolkit. In: Proceedings of 52nd annual meeting of the association for computational linguistics: system demonstrations, 2014; p. 55–60.

[48] Hamon T, Grabar N (2010). Linguistic approach for identification of medication names and related information in clinical narratives. J Am Med Inform Assoc.

[49] Wu Z (2020). A comprehensive survey on graph neural networks. IEEE Trans Neural Netw Learn Syst.

[50] Kipf TN, Welling M. Semi-supervised classification with graph convolutional networks. In: Proceedings of the 5th International Conference on Learning Representations (ICLR). 2017.

[51] Chen P et al. Explicitly capturing relations between entity mentions via graph neural networks for domain-specific named entity recognition. In: Proceedings of the 59th Annual Meeting of the Association for Computational Linguistics and the 11th International Joint Conference on Natural Language Processing (Vol. 2). 2021; p. 735–742.

[52] Tran T (2020). Syntactically-informed word representations from graph neural network. Neurocomputing.

[53] Robinson JJ. Dependency structures and transformational rules. Language. 1970; p. 259–285.

[54] Smith L (2008). Overview of BioCreative II gene mention recognition. Genome Biol.

[55] Kim J-D et al. Introduction to the bio-entity recognition task at JNLPBA. In: Proceedings of the international joint workshop on natural language processing in biomedicine and its applications, 2004; p. 70–75.

[56] Pafilis E (2013). The SPECIES and ORGANISMS resources for fast and accurate identification of taxonomic names in text. PLoS ONE.

[57] Gerner M (2010). LINNAEUS: a species name identification system for biomedical literature. BMC Bioinformat.

[58] Li J (2016). BioCreative V CDR task corpus: a resource for chemical disease relation extraction. Database.

[59] Doğan RI (2014). NCBI disease corpus: a resource for disease name recognition and concept normalization. J Biomed Inform.

[60] Krallinger M (2015). The CHEMDNER corpus of chemicals and drugs and its annotation principles. J Cheminformat.

[61] Kanerva J (2020). Dependency parsing of biomedical text with BERT. BMC Bioinformat.

